# Method for Passive Droplet Sorting after Photo-Tagging

**DOI:** 10.3390/mi11110964

**Published:** 2020-10-28

**Authors:** Chandler Dobson, Claudia Zielke, Ching W. Pan, Cameron Feit, Paul Abbyad

**Affiliations:** Department of Chemistry and Biochemistry, Santa Clara University, Santa Clara, CA 95053, USA; cdobson@scu.edu (C.D.); czielke@scu.edu (C.Z.); chingwei_pan@urmc.rochester.edu (C.W.P.); cfeit@scu.edu (C.F.)

**Keywords:** microfluidics, droplet microfluidics, sorting, passive sorting, photo-tag, droplet array

## Abstract

We present a method to photo-tag individual microfluidic droplets for latter selection by passive sorting. The use of a specific surfactant leads to the interfacial tension to be very sensitive to droplet pH. The photoexcitation of droplets containing a photoacid, pyranine, leads to a decrease in droplet pH. The concurrent increase in droplet interfacial tension enables the passive selection of irradiated droplets. The technique is used to select individual droplets within a droplet array as illuminated droplets remain in the wells while other droplets are eluted by the flow of the external oil. This method was used to select droplets in an array containing cells at a specific stage of apoptosis. The technique is also adaptable to continuous-flow sorting. By passing confined droplets over a microfabricated trench positioned diagonally in relation to the direction of flow, photo-tagged droplets were directed toward a different chip exit based on their lateral movement. The technique can be performed on a conventional fluorescence microscope and uncouples the observation and selection of droplets, thus enabling the selection on a large variety of signals, or based on qualitative user-defined features.

## 1. Introduction

Droplet microfluidics confines chemicals or cellular reagents in picoliter droplets transported in inert oil and is well suited for large-scale chemical or biological analysis [1,2]. The selection of droplets is a basic operation for applications such as cell screening, [3,4] enzyme directed evolution [5], and metabolic engineering [6,7]. In active sorting, [8] droplets that exceed a fluorescence signal threshold, are synchronized with an electric field, [9,10] magnetic field, [11] acoustic wave, [12] pneumatic pressure [13], or light [14] to direct selected droplets to a predetermined channel exit. For example, fluorescence-activated droplet sorting (FADS) [10] uses electric fields for robust selection at rates as high as 30 kHz [15].

Although powerful, these techniques are endpoint measurements that require the observation and active sorting to be performed close to each other in both time and space. Droplets are observed in series and the order of droplets must be maintained to ensure that the active sorting component selects the correct droplets. This limits the sorting criteria in two major ways. The fluorescence signals must generally be measured just prior to active sorting. Therefore, droplets cannot be selected based on the time evolution of signals or signals that would develop or saturate much earlier in time. Second, in-line sorting also limits the observation time of droplets to approximately the droplet sorting time. For fast sorting, the selection signal is based on moving droplets integrated for milliseconds or less. The signal must be easily interpretable without user intervention. Selection is thus primarily based on fluorescence signals, [10,16] and, in rare occasions, fluorescence lifetime [17] and absorbance [18]. It excludes weak signals that would require longer integration or signals that would require specialized equipment or user interpretation. Examples of techniques that would be difficult to integrate with active droplet sorting due to a combination of instrumentation and long signal integration include FT-IR [19] and Raman detection [20], UV-Vis absorbance, fluorescence anisotropy, [21], and droplet radiofluidics [22]. Cell selection based on subtle differences in cell morphology, important criteria for determining cell type and state, often requires user interpretation. Kinetic measurements in droplets, such as fast kinetics [23] and Michaelis-Menton kinetic measurements [24], are also difficult to integrate with current active droplet sorting technology.

Droplet arrays [25,26,27] provide a platform for extended observation of droplets with selection either by mechanical means with pneumatic valves [28] or via photoactivation, producing an air droplet that selectively pushes the droplet out of the well [29,30]. Both methods have potential drawbacks. Valves increase the complexity and control elements of the device. Photoactivation requires a UV or NIR laser and can cause localized heating that may damage droplet content. Moreover, as selection is based on droplet location or address in the array, both the droplet observation and selection must be performed on the array. This can limit the type of operations or steps that can be performed on the droplets.

We present here a simple and inexpensive method that can be performed on a conventional fluorescence microscope that uncouples the observation and selection of droplets allowing the selection on a larger variety of signals.

We have recently developed a passive technology, Sorting by Interfacial Tension (SIFT), [31,32] that sorts droplets based on differences in interfacial tension that is linked to droplet pH. The technique was used previously to sort droplets containing enzymes [31] and cells [32,33]. In both these cases, the change in the pH was due to processes incurred by droplet content not user control. In the case of enzymes, the change of pH was from the production of an acidic byproduct. For cells, the release of protons associated with glycolysis induced a pH change. Hence, these applications did not allow the selection of an arbitrary droplet by the user.

Photoacids have the property of becoming more acidic upon light excitation. Droplets containing the photoacid, 8-hydroxypyrene-1,3,6-trisulfonic acid trisodium salt (HPTS), henceforth called by its common name pyranine, were excited, leading to a decrease in pH and a concurrent increase in droplet interfacial tension. This provides a method to “tag” droplets of interest for latter passive sorting based on differences in interfacial tension.

The “tagging” of droplets for selection uncouples the observation step from the sorting step. This is a departure from most sorting techniques, such as FADS, where the observation and sorting are required to occur close together in both time and space. The observation step can be as long as needed. This opens the door for selection of droplets from signals that require specialized equipment, long integration time, or user intervention (i.e.,: absorbance, kinetic data, cell morphology, IR, Raman, fluorescence lifetime, anisotropy, and droplet radiofluidics). The technique is easy to perform and requires only a conventional fluorescence microscope. It is flexible as the tagging can be performed anywhere on the chip. This simplifies device design and the integration of measurement and sorting within the same chip. The technique does not require the detector, active sorting element, and related electronics of FADS. Thus, it can greatly decrease the cost and complexity of an analysis.

The mechanism and application of the sorting method are presented here. First, the change in pH of droplets as a function of the duration of light excitation is characterized. The technique is used to select individual droplets within a droplet array. Spatial separation is demonstrated by exciting or tagging specific droplets long before the downstream passive sorting of these same droplets. The technique is also used to select desired cells in a droplet array based on user-determined apoptosis stage. By uncoupling the measurement and sorting of droplets, this novel method presents a simple way to increase the variety and complexity of signals that can be used as the basis for droplet selection.

## 2. Materials and Methods

### 2.1. Microfluidic Device

Polydimethylsiloxane (PDMS) microfluidic chips with channel depth modulations were fabricated using the dry-film photoresist soft lithography technique described by Stephan et al. [34]. The technique enabled rapid prototyping of multi-level structures. The PDMS chips were then plasma-bonded to a glass slide. To render the internal channel surface hydrophobic, Novec™ 1720 Electronic Grade Coating (3M) was flowed into the microchannel and the chip was heated for 30 min at 150 °C. The surface treatment prevented wetting and contact of the aqueous droplets with the channel walls.

### 2.2. Beads 

A solution of 2 µm fluorescent red beads (Sigma-Aldrich, Milwaukee, WI, USA) in a 1:20,000 ratio was prepared in 2.5 mM PBS buffer supplemented with Optiprep (15% *v*/*v*, Fresenius Kabi Norge AS for Axis-Shield PoCAS, Oslo, Norway), Pluronic F-68 (1% *w*/*w*, Affymetrix Inc., Maumee, OH, USA), and 2 mM pyranine (AAT Bioquest Inc., Sunnyvale, CA, USA). Optiprep was added to limit bead and cell sedimentation in the tubing and droplets, whereas Pluronic F-68 increased cell viability and droplet stability. Pyranine is a photoacid and used in these experiments to selectively mark then sort droplets. In addition, it serves as fluorescent ratiometric probe for pH analysis of droplets, based on a calibration curve of droplets of known pH.

### 2.3. Cells 

Jurkat, Clone E6-1 (ATCC TIB-152, human acute T-cell leukemia) cells were grown in ATCC-formulated RPMI-1640 Medium, containing 10% fetal bovine serum (HyClone, GE Healthcare Life Sciences, Logan, UT, USA) and 1% penicillin-streptomycin (Gibco, Life Technologies Corporation, Grand Island, NY, USA). The growing conditions were 37 °C and 5% CO_2_.

A population of Jurkat cells was harvested through centrifugation, washed with PBS, and resuspended in PBS. The harvested cells were kept for 1–3 days on the bench to induce apoptosis. On the day of experiment, cells were centrifuged, resuspended in 2.5 mM PBS buffer supplemented with Optiprep, Pluronic F-68 and pyranine as described above. Both, pH and osmolality (determined with Vapro Vapor Pressure Osmometer 5520, Wescor, ELITech Biomedical Systems, Logan, UT, USA) of the solutions were analyzed and adjusted to match physiological values (pH 7.4; 280–320 mOsmol). Around 2 drops/mL of Invitrogen Propidium Iodide ReadyProbes™ Reagent (Fisher Scientific) were added to visualize apoptosis based on membrane integrity under fluorescent light.

On the day of experiment, a second population of Jurkat cells was harvested and prepared for on-chip experiments following the same procedure as described above. Both apoptotic and freshly harvested cells were mixed in a 1:1 ratio before injection onto the chip to ensure a variety of cell viability stages for the experiment. To reduce the amount of double occupied droplets, the cell density was analyzed with a Cellometer Auto T4 Bright Field Cell Counter (Nexcelcom Bioscience LLC, Lawrence, MA, USA) and adjusted to about 1 × 10^6^ cells/mL prior to injection.

### 2.4. Measurements 

The temperature of the chip was maintained at 37 °C using a microscope heating stage with control module with temperature feedback (CHS-1 heating plate with TC-324C temperature controller, Warner Instruments). Fluid flow was controlled using computer-controlled syringe pumps (Nemesys, Cetoni). Droplets were produced in 0.1% Picosurf in perfluorinated oil, Novec 7500 (Sphere Fluidics, Cambridge, UK). Another perfluorinated oil with dissolved surfactant, Droplet Generation Oil for Probes (Bio-Rad, Hercules, CA, USA), hereby called QX100, was used to push out droplets from the array and within the sorting rail region.

Images and videos were taken on an inverted fluorescence microscope (Olympus IX-51) with a shuttered LED fluorescence excitation source (Spectra-X light engine, Lumencor, Beaverton, OR, USA) and a high-speed camera (VEO-410, Vision Research, Wayne, NJ, USA). The excitation source had individual addressable LEDs that included violet (395 nm BP 25 nm), blue (436 nm BP 28 nm), and green (561 nm BP 14 nm) light. Based on manufacturer specifications, each LED had approximately 300 mW of power. The excitation source was coupled to an Arduino (Arduino LLC, Scarmagno, Italy) to rapidly alternate between different colored LEDs or to use two simultaneously using simple TTL triggering. For photo-tagging droplets, a 40× objective was used with a partially closed iris to limit the area of excitation. The microscope filter cube contained a dual-edge dichroic mirror (Di03-R488/561-t1-25 × 36, Semrock, IDEX Health & Science LLC Rochester, NY, USA) and dual-band emission filter (FF01-523/610-25, Semrock) that enabled transmission of both pyranine and the red fluorescence of the beads or propidium iodide.

Droplets containing fluorescent beads were identified using green excitation light. Cell apoptosis stage was identified using a 40× magnification. Live cells (spherical) and necrotic cells (decomposed) were identified by morphology using bright field. Late apoptosis cells were distinguished from early apoptosis cells by propidium iodide fluorescence using green excitation. Images and videos were analyzed using ImageJ [35].

## 3. Results and Discussion

In droplet microfluidics, reagents are encapsulated in aqueous droplets transported in inert oil enabling increases in throughput in applications such as digital PCR, directed evolution, drug screening, and single-cell analysis [4,5,10,16]. A critical step for many applications is the selection and recovery of droplets of interest. Sorting is performed by first observing the droplet, then using an active component to direct desired droplets to a different chip exit.

We have recently developed a label-free technique to sort droplets based on pH. The technique is based on the observation that the interfacial tension of droplets can be very sensitive to droplet pH for a specific oil and surfactant combination. In particular, for droplets in the external oil and surfactant combination of QX100, interfacial tension increases from 12 to 24 dyn/cm as pH decreases from 8 to 6 [32]. These pendant-drop measurements were performed with QX100 solution diluted 100-fold with perfluorinated oil. Droplets of undiluted QX100 quickly detached from the needle. The interfacial tension of droplets in undiluted QX100, as used in experiments, is much lower and estimated to be a few dyn/cm from calculations [31]. The source of the pH sensitivity of QX100 is suspected to be due to a change in protonation state of the surfactant; however, the proprietary nature of the product renders it difficult to confirm the exact mechanism [31].

In a technique dubbed Sorting by Interfacial Tension (SIFT), differences in interfacial tension of droplets provides a handle to sort droplets based on pH. The method utilizes flattened droplets, confined by the top and bottom of the channel. Droplets encounter a microfabricated trench, or rail, of increased channel height (rail geometry in Supplemental Figure S1). The droplets expand into the rail, decreasing their surface area and energy. To leave the rail, droplets need to be squeezed again. Droplets of low interfacial tension enter the rail but are pushed off due to the drag of the external oil flow. The oil flow is insufficient to push droplets of higher interfacial tension off the rail. These droplets follow the tapered rail oriented at 45 degrees relative to the direction of flow. These droplets leave at a different lateral position as compared to droplets of low interfacial tension. Using different channel exits, droplets of different interfacial tension and hence pH are sorted. The technique has been utilized for label-free sorting of different enzymes based on activity [31] and of cells based on glycolysis [32,33]. In these applications, the pH change was induced by droplet content rather than external control.

The SIFT technique is expanded here by using light to induce a selective pH change in a droplet containing a fluorescent dye. The fluorophore pyranine is a water-soluble dye that is used as a staining agent, pH indicator, and model system for excited-state proton transfer (ESPT) [36,37]. It is cell membrane impermeant and considered biocompatible [38]. When excited, pyranine gives off a characteristic yellow-green fluorescence. It is a ratiometric pH indicator as the excitation spectrum is modulated by pH, shifting from blue (440 nm) to violet (405 nm) with decreasing pH [39].

**Figure 1:**
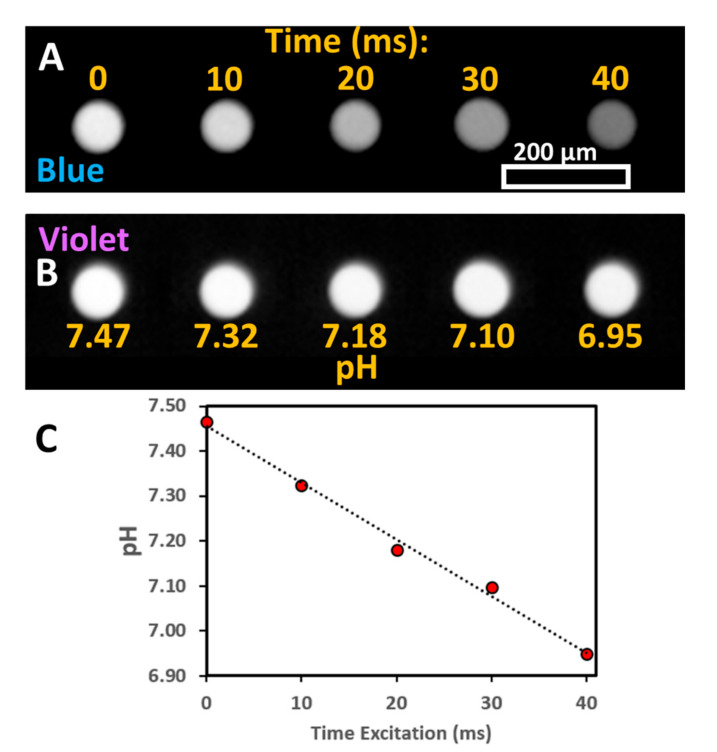
Linear array of droplets with increasing irradiation times from left to right. (**A**) Fluorescence from blue excitation (436 nm). (**B**) Fluorescence from violet excitation (395 nm). pH of droplets as determined from blue to violet fluorescence intensity ratio based on calibration curve as shown in Supplemental Figure S2. (**C**) Droplet pH with increasing irradiation time with fit to a linear regression.

It was observed that the excitation of a solution of pyranine leads to a decrease in solution pH as measured with a pH meter. No change in pH was observed in the absence of pyranine. The change in pH was characterized as a function of irradiation time with light for a linear array of stationary droplets (Figure 1). The droplets were irradiated simultaneously with both violet (395 nm BP 25 nm) and blue (436 nm BP 28 nm) light using a 40× objective. After irradiation, the fluorescence was monitored with either blue or violet light with a 10× objective. For the blue excitation channel (Figure 1A), the fluorescence intensity decreases as a function of irradiation time. In contrast, for violet excitation (Figure 1B), the fluorescence intensity remains mostly constant with irradiation time.

The pH of droplets after irradiation can be determined by the ratio of blue to violet excitation of pyranine using a calibration curve (Supplemental Figure S2). The decrease in pH is correlated with irradiation time (Figure 1C) and is observed to be mostly linear. For long irradiation times (>100 ms), the ratio of violet to blue was no longer proportional to excitation time. It was found that the change of pH was faster with the irradiation of droplets with a combination of violet and blue LED lights than either alone (data not shown). The monitoring of droplet pH could itself induce a pH change. To limit this effect, the exposure time was limited and a lower numerical aperture objective was used for monitoring (10× magnification with a numerical aperture of 0.30) than for irradiation (40× magnification with a numerical aperture of 0.60).

It is worth noting that the ratio of violet to blue fluorescence for a given pH was the same regardless of whether a change in pH was induced by light or the addition of a strong acid (Supplemental Figure S3). This shows that pyranine can serve both to induce a pH change as well as to report droplet pH.

As a photoacid, pyranine shows a marked increase in acidity in the excited state with a pKa that decreases from 7.4 to 0.4 in the excited state [40]. Thus, the photoexcitation of pyranine would lead to a release of a proton. This proton would be expected to return upon relaxation to the ground state. However, intense excitation can lead to photooxidation and radical reactions of the pyranine in the excited state, [41,42] and lead to the observed change in droplet pH. The destruction of fluorophore is consistent with the constant fluorescence intensity upon violet excitation (Figure 1B) as an increase would otherwise be expected at lower pH for a constant pyranine concentration.

**Figure 2:**
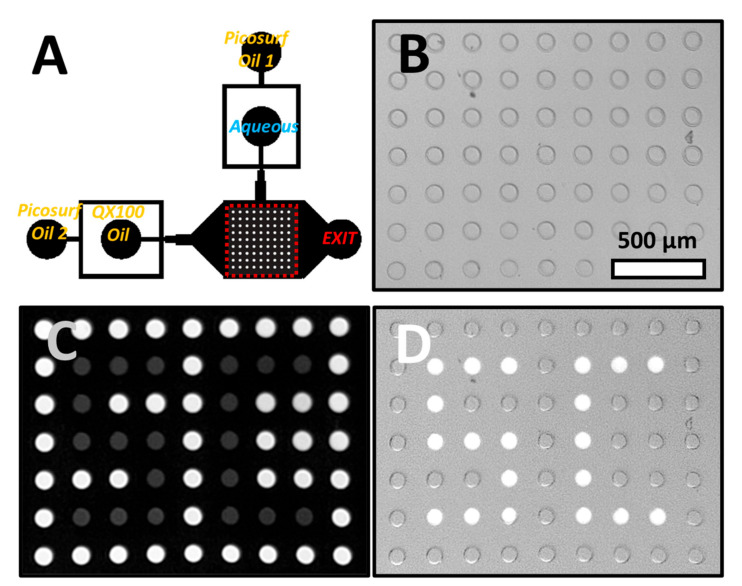
(**A**) Array device channel geometry. The location of the droplet array is highlighted by a dashed red square. (**B**) Bright-field image of droplet array. All wells are occupied with droplets that are approximately the same size as the wells. (**C**) Fluorescence from blue excitation. Irradiated droplets have lower fluorescence intensity. (**D**) Droplet array after the elution of non-irradiated droplets. Fluorescence excitation light was used in combination with bright-field image to increase droplet visibility.

The decrease in droplet pH upon irradiation can be used to select specific droplets of a droplet array as shown in Figure 2 and Video S1. Figure 2A shows the channel geometry as well as the inlets and outlets of the microfluidic device. The droplets at an initial pH of 7.46 were anchored in an array of wells using a technique called Rails and Anchors, [26] where confined droplets expand into wells (Figure 2B). Droplets were approximately the size of the wells. To exit the well, droplets need to be squeezed from their height in the well (50 µm) to the height of the channel (25 µm). In the presence of QX100, interfacial tension increased with decreasing droplet pH. Therefore, droplets of higher pH are pushed out of the wells at lower external flow rates (Supplemental Figure S4). The flow required for droplet ejection also provided an estimate of droplet pH. This was useful for longer irradiations and lower pH when ratiometric readings of pyranine fluorescence did not provide an accurate estimate of droplet pH.

Selected droplets were irradiated for about a second as described above with violet and blue light using a 40× objective. This excitation led to a decrease of over 1 pH unit as estimated from the external flow required for elution. The droplets were noticeably dimmer when excited with blue light (Figure 2C). Next, QX100 was flowed at high flow rates, 80–100 µL/min, into the device. The droplets at higher pH were quickly displaced from the wells. However, for the irradiated droplets at lower pH and hence higher interfacial tension, the hydrodynamic drag was insufficient to push out the droplets. These droplets remained in the wells as seen in the fluorescence image in Figure 2D (letters “S” and “C” produced with droplets). Increasing the flow to a much higher rate, typically around 150 µL/min, caused the elution of all droplets in the array. The photoselection of droplets was dependent on both the presence of pyranine in the droplet and the use of QX100 oil. The absence of either led to no correlation between droplet excitation and droplet elution (Supplemental Figure S5).

By the general method described above, the observation and selection can be uncoupled in time. Droplets can be observed and studied in the array for an arbitrary length of time. The droplets of interest can then be selected, either by irradiation to remain in the array (positive selection) or through elution (negative selection) by irradiating undesired droplets.

**Figure 3:**
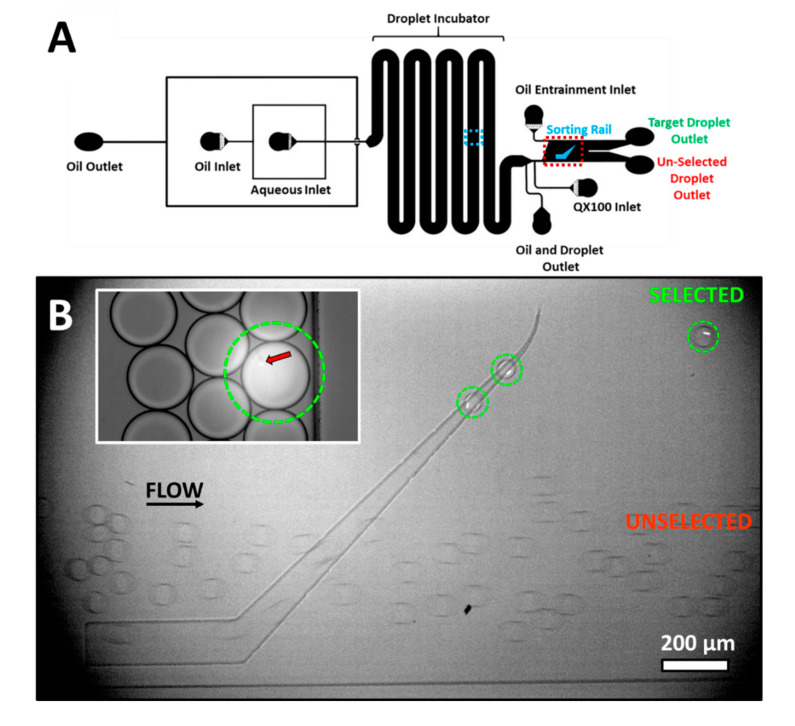
(**A**) Rail device channel geometry. The location of the images in (**B**) are shown. Location of droplet irradiation is indicated by a blue dashed rectangle and the sorting rail is highlighted by a dashed red rectangle. (**B**) Droplets containing fluorescent beads, circled in green, were previously irradiated with light. Irradiated droplets have lower pH and hence higher interfacial tension. They follow the rail upwards and leave at a higher lateral position toward the selected exit. Other droplets are immediately pushed off the rail by the flow of oil toward the unselected exit. Inset: A droplet circled in green was irradiated prior to sorting. Neighboring droplets are also partially irradiated. The red arrow indicates a fluorescent bead.

A similar technique can also be used to separate the observation and selection in space, each performed in different regions of a microfluidic device. In contrast to the use of a droplet array, this variant, using a microfabricated rail (Figure 3A), would be more amenable to continuous sorting rather than batch processing. As a demonstration, droplets were produced of which a small percentage, <2%, contained fluorescent beads. Flow was slowed and droplets containing beads were selectively irradiated for 1 s as shown in Figure 3B, inset. This illumination induced a decrease in pH of about 1 unit from the initial pH of 7.4 based on a similar exposure in the droplet array device. As visible from the image, the illumination area was slightly larger than the droplet leading to the slight irradiation of the neighboring droplets. However, this off-target illumination was insufficient to lead to a sizable change in pH or to influence sorting.

Approximately 90–120 s after irradiation, illuminated droplets were flowed to the sorting region. The sorting region was located over 2 cm downstream from the location of droplets irradiation. As the droplets enter the sorting region, QX100 is introduced into the channel. The external oil flow and hence the drag can be controlled in the sorting region. This control is provided by an additional QX100 inlet, denoted as the Oil Entrainment Inlet in Figure 3A. Supplemental Table S1 provides typical flow rates in the device. Figure 3B and Video S2 show the sorting of droplets containing beads. Empty droplets, that were not irradiated, enter the sorting rail but are quickly pushed off by the entrainment flow and exit the chip via the unselected exit. The irradiated droplets containing beads have higher interfacial tension in the presence of QX100. The external flow of oil is insufficient to push the droplets off the rail. The droplets followed the tapered rail upwards leaving near the end point. These droplets (circled in green) exit the chip via the selected exit.

The method is able to isolate photo-tagged droplets within a large excess of droplets. In this case, a large change in pH was induced by light to ensure clearly differentiated droplet populations. However, the entrainment flow provides an independent user-defined parameter that can be finely tuned to select between droplets with smaller pH differences. For example, droplets irradiated for 50 ms and with a pH difference of 0.4 units were separated using the device as shown in Supplemental Figure S6. When droplets come in contact with QX100, they become more acidic as revealed by the dimmer droplets with blue excitation near the rail. This is likely because the surfactant in QX100 is acidic, however the proprietary nature of the product makes this difficult to confirm.

As a demonstration of an application of the technique, cells contained in droplets were selected on the basis of their stage in apoptosis through visual characterization, without fixing the cells. Cell apoptosis is programmed cell death, which is a vital part of biological cell turnover and an important process to ensure proper development. Improper apoptosis is linked to many disease states [43]. Apoptosis can be identified through distinct morphological changes of a cell. Using the technique presented, different cell states (viable, early apoptosis, late apoptosis, necrosis) can be observed, evaluated, selected, and collected for further analysis.

**Figure 4:**
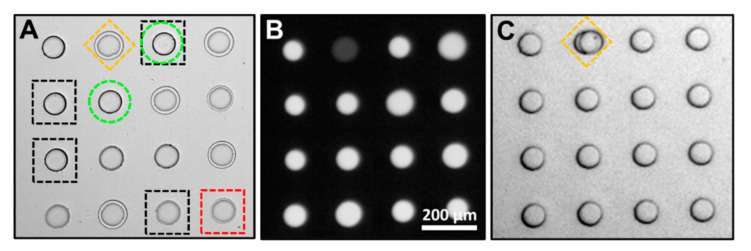
(**A**) Bright-field image of droplet array. Droplets containing cells were classified as viable (green circle), early apoptosis (yellow diamond), late apoptosis (red square), and necrosis (black square). (**B**) Fluorescence image with blue excitation. The droplet with an early apoptosis cell was irradiated and displays lower fluorescence intensity. (**C**) Droplet array after the elution of non-irradiated droplets. The only droplet remaining in the array contains the early apoptosis cell.

Using the device shown in Figure 2A, cells at different stages of cell death were encapsulated in droplets and anchored within an array. The cells were then evaluated and categorized (viable, early apoptosis, late apoptosis, necrosis) by user observation as described in the Materials and Methods (Figure 4A). A droplet containing a cell identified as early apoptosis (yellow diamond) was illuminated with light leading to a decrease in pH from 7.4 to 6.5. This change in pH can be observed as a decrease in fluorescence signal when excited with blue light (Figure 4B). QX100 was introduced into the chip at a flow rate of 120 µL/min ejecting empty droplets and droplets containing cells of different apoptosis stages from the array. The sole remaining droplet in the array contains the photo-tagged droplet containing the early apoptosis cell. This selected droplet can be ejected by increasing the flow rate even higher to 140 µL/min.

The method presents a way to select droplets and hence cells of interest. Here, the droplet with the cell of interest was irradiated and remained in the array. Alternatively, the undesired droplets can be irradiated with the desired droplets ejected. Depending on the application, this may be preferable as the excitation and decrease of the pH could damage cells. This would also limit exposure to the surfactant, QX100, that may have limited biocompatibility [32,44]. For the collection of multiple cell types, several illumination times could be used to induce different pH changes. This would allow for sequential elution of droplets by increasing flow rates.

## 4. Conclusions

We present a method to photoselect and then recover individual droplets by passive sorting. It can be used to select droplets in a droplet array or to direct photo-tagged droplets toward a different chip exit using a microfabricated rail. The technique is simple and inexpensive to adapt as it requires only a conventional fluorescence microscope without the need for the detector, active sorter, and related electronics used for fluorescence-activated droplet sorting (FADS). The technique uncouples the observation and sorting of droplets, thus facilitating the selection based on signals that are difficult to adapt to FADS including weak signals, kinetic data, long integration times, or qualitative features.

The method presented here can be adapted for efficiency and sensitivity. With the LED excitation source used, excitation times required here for sorting were as little as 50 ms. This time can likely be decreased with a more intense laser source. Likewise, both lowering the buffering capacity or increasing the pyranine concentration of solution will also decrease the excitation times. Pyranine, with an excitation spectrum that spans up to 500 nm, may interfere with some assays. Other photoacids, [45] could possibly be used instead to provoke a light-induced pH change. Ideal droplet tagging would cause a minimum change or perturbation to the droplet. In this initial demonstration, the change in pH was large which allowed for robust selection. However, by carefully tuning the flow conditions, consistent selection can be performed based on small pH differences, as little as 0.05 units [33]. Excitation position was adjusted here by hand using the microscope stage. A computer-controlled excitation source, using galvanometric mirrors, [46] could allow more precise and automated droplet tagging.

## Supplementary Materials

Supplementary materials can be found at https://www.mdpi.com/2072-666X/11/11/964/s1.
