# Supplementary material for: Method for Passive Droplet Sorting after Photo-Tagging
Source: Micromachines (Basel). 2020 Oct 28;11(11):964. doi: 10.3390/mi11110964 (PMC7692103; doi:10.3390/mi11110964)

micromachines

Article

Method for Passive Droplet Sorting After Photo-Tagging

Chandler Dobson †, Claudia Zielke †, Ching W. Pan, Cameron Feit and Paul Abbyad *

Department of Chemistry and Biochemistry, Santa Clara University, Santa Clara, CA 95053, USA; cdobson@scu.edu (C.D.); czielke@scu.edu (C.Z.); chingwei_pan@urmc.rochester.edu (C.W.P.); cfeit@scu.edu (C.F.)

* Correspondence: pabbyad@scu.edu

† Both authors contributed equally to this work.

Received: 21 September 2020; Accepted: 26 October 2020; Published: date

Supplemental Information

Table of Contents:

Video S-1………………………………………………………………………..…...Page S-2

Video S-2………………………………………………………………………..…...Page S-2

Figure S-1……………………………………………………………………..…...Page S-3

Figure S-2……………………………………………………………………..…...Page S-4

Figure S-3……………………………………………………………………..…...Page S-5

Figure S-4……………………………………………………………………..…...Page S-6

Figure S-5……………………………………………………………………..…...Page S-7

Figure S-6……………………………………………………………………..…...Page S-8

Table S-1………………………………………………………………………..…...Page S-9

Video Caption:

Video S1. Demonstrates the steps to select droplets within an array. Desired droplets are first irradiated before eluting the non-irradiated droplets in the array using the flow of QX100 oil.

Video S2. Droplets containing fluorescent beads were previously irradiated with light. Irradiated droplets have lower pH and hence higher interfacial tension. They follow the rail upwards and leave at a higher lateral position towards the Selected exit. A large number of empty non-irradiated droplets are immediately pushed off the rail by the flow of oil towards the Unselected outlet. One droplet containing a bead was not irradiated. It is pushed off the rail and exits from the Unselected outlet

.

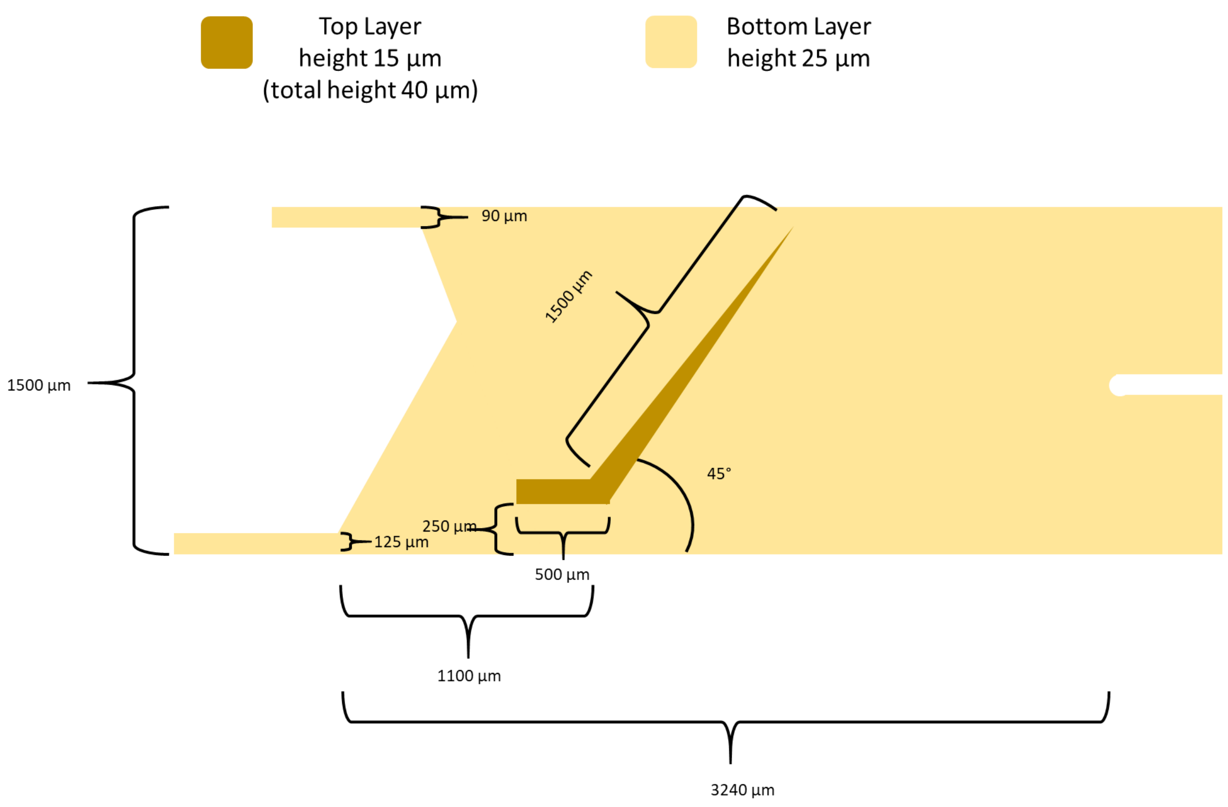

Supplemental Figure S1. Sorting rail dimensions and position. Exact position of rail is approximate as layers are positioned by eye.

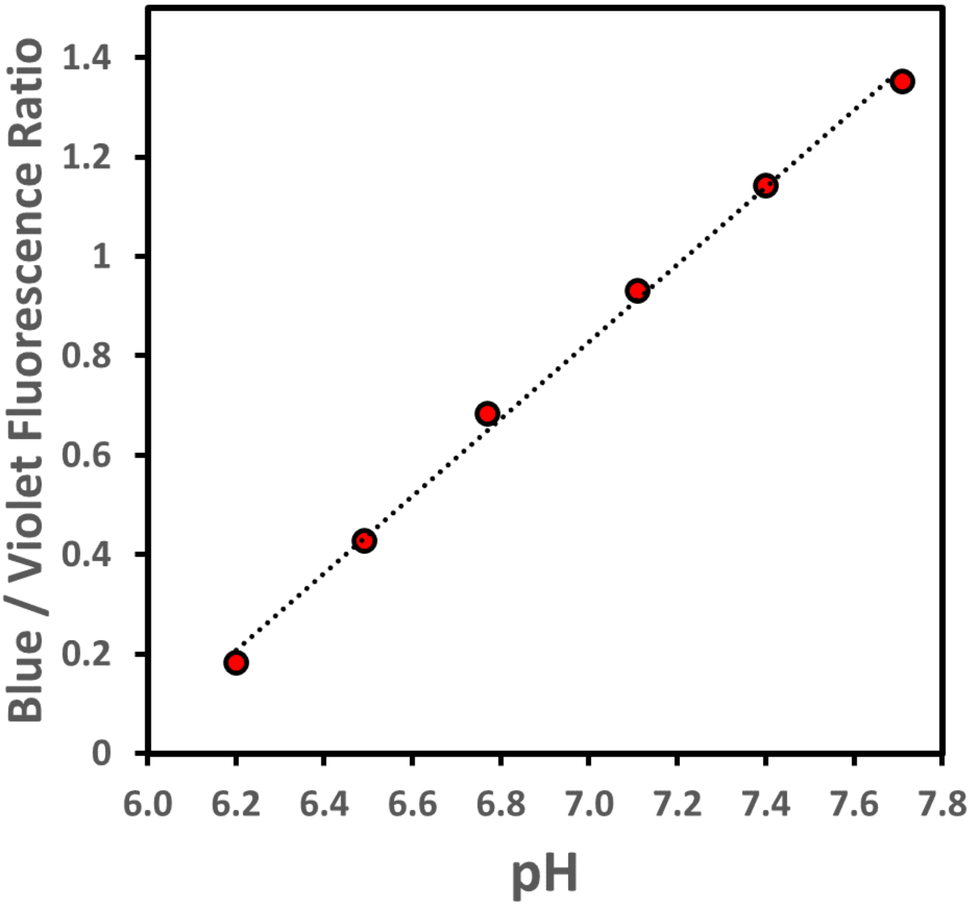

Supplemental Figure S2. Blue to violet fluorescence intensity ratio vs pH of droplets containing pyranine, a pH sensitive ratiometric fluorescent probe. Solutions of known pH were injected into the microfluidic device and droplets were analyzed for their normalized fluorescence intensity through excitation with blue (436 nm) and violet (395 nm) light. The slope of the calibration curve was found to be consistent from day to day, whereas the y-intersection was adjusted for each experiment using the known pH of empty droplets.

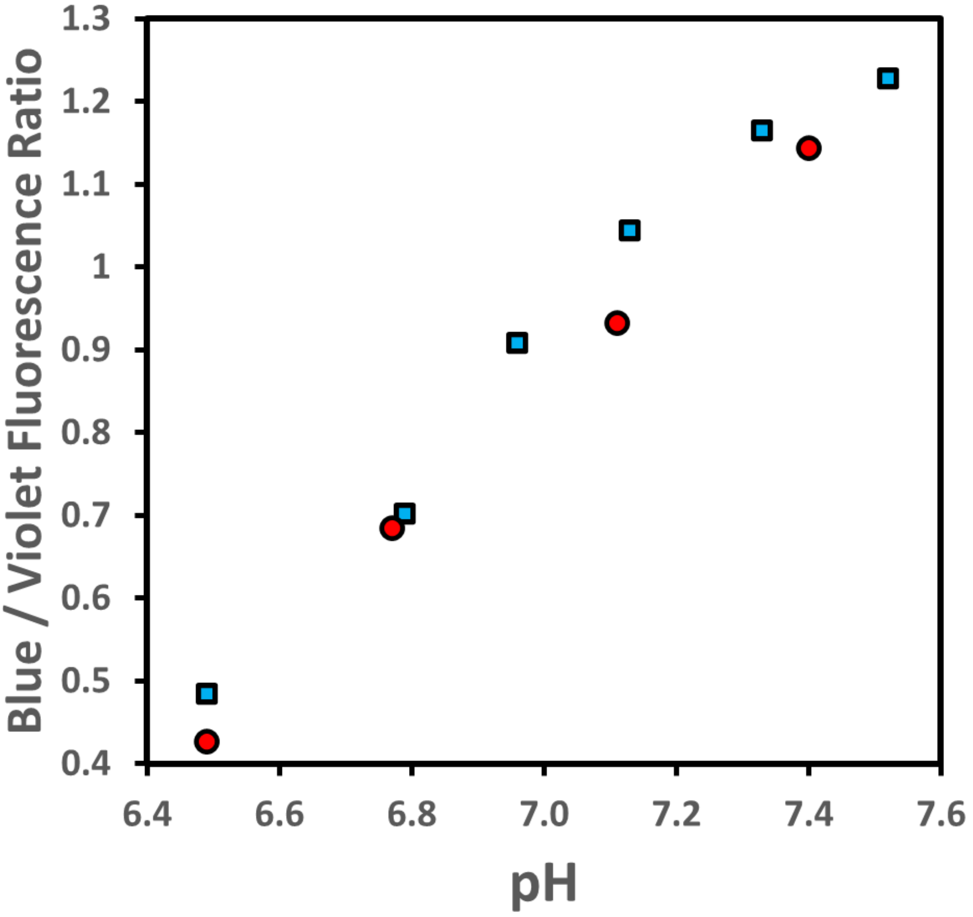

Supplemental Figure S3. Blue to violet fluorescence intensity ratio vs pH of droplets where the pH change was induced by irradiation with light (blue squares) or with acid (red circles). Solution was irradiated with light off-chip in a microcentrifuge tube. The pH was measured with a pH meter before injecting the solutions into the microfluidic device to produce droplets. The fluorescence of droplets was measured for excitation with blue (436 nm) and violet (395 nm) light.

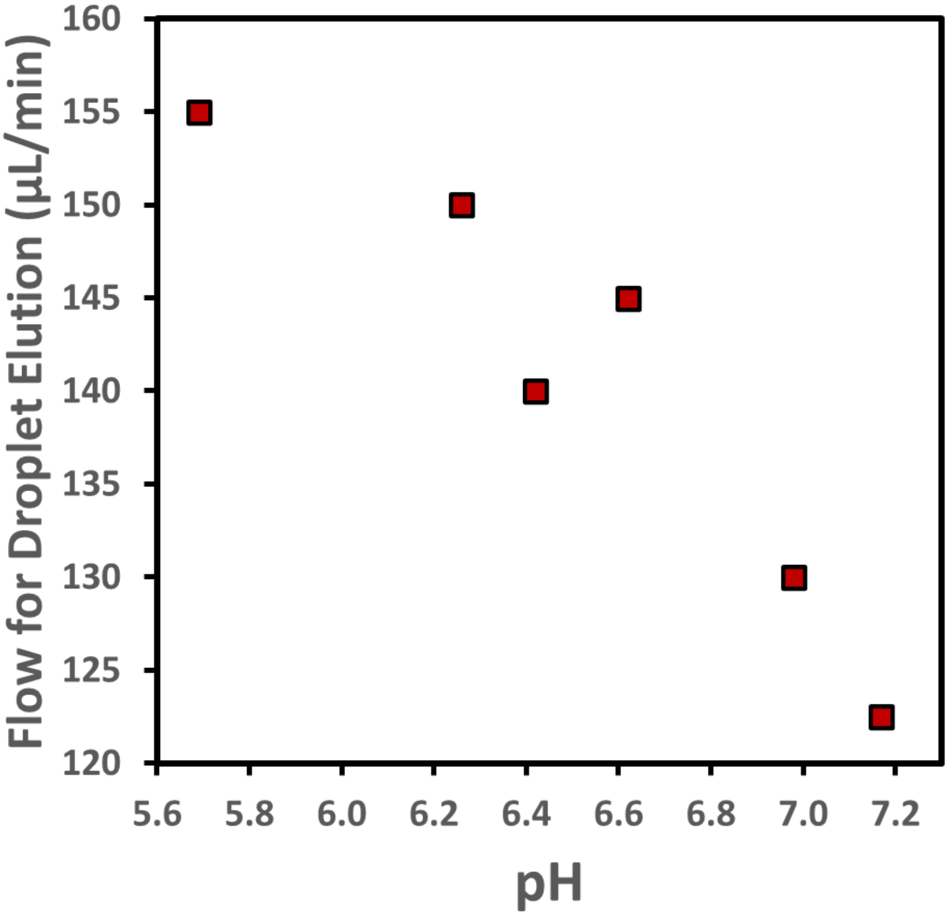

Supplemental Figure S4. External oil flow rate required to elute droplets from the wells with QX100 in the droplet array device. Channel geometry shown in Figure 2a.

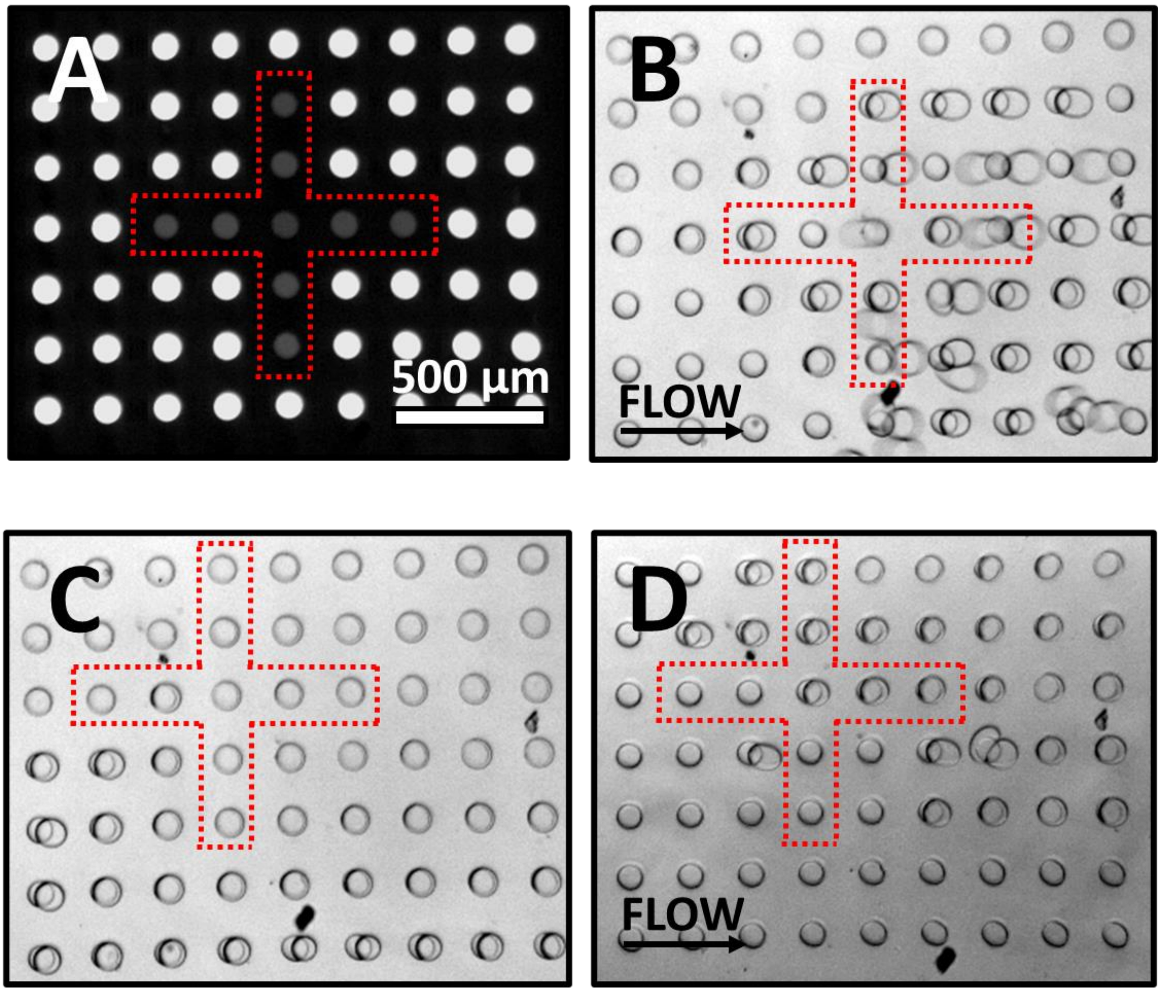

Supplemental Figure S5. Control experiments showing elution of droplets after irradiation for about a second in the absence of QX100 (a,b) and pyranine (c,d). **a)** Fluorescence image with blue excitation. Irradiated droplets are darker and are indicated by the red dotted cross. **b)** Elution of droplets by 0.1% Picosurf in Novec 7500. Irradiated droplets do not show a higher tendency to remain in the well. Several irradiated droplets have been pushed out of wells. More droplets occupy wells on the right of image as eluted droplets will get temporarily captured by downstream wells. **c)** Droplets array in the absence of pyranine. Irradiated droplets are indicated by red dotted cross. **d)** Irradiated droplets do not show a higher tendency to remain in the well. Several irradiated droplets have been pushed out of wells.

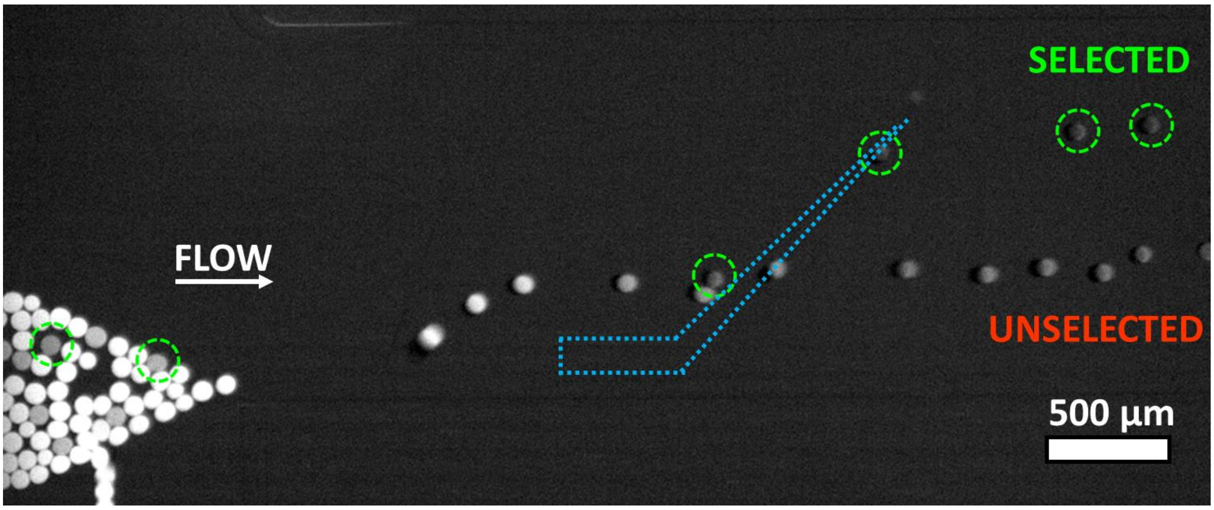

Supplemental Figure S6. Fluorescence image with blue excitation of the sorting of two populations of droplets using a rail. A few droplets were irradiated for 50 ms prior to sorting that induced a change of pH from 7.4 to 7.0 as determined by blue to violet fluorescence ratio. The irradiated droplets appear dimmer in the image. Irradiated droplets follow the rail upwards and leave at a higher lateral position towards the Selected exit (a few droplets circled in green). Other droplets are immediately pushed off the rail by the flow of oil towards the Unselected exit. The position of the rail is outlined in blue.

Supplemental Table S1. Typical flow parameters used in experiments. Channel geometry is provided below for reference.

Inlets and Outlets	Flow Rates (µL/min)
Aqueous Inlet	0.1 - 0.5, most commonly 0.3
Oil Inlet	3 – 5, most commonly 3
QX100 Inlet	2 – 5
Oil Entrainment Inlet	8 – 20
Oil Outlet	- 2 to -3 (NEGATIVE FLOW)
Oil and Droplet Outlet	-0.25 to -1 (NEGATIVE FLOW)

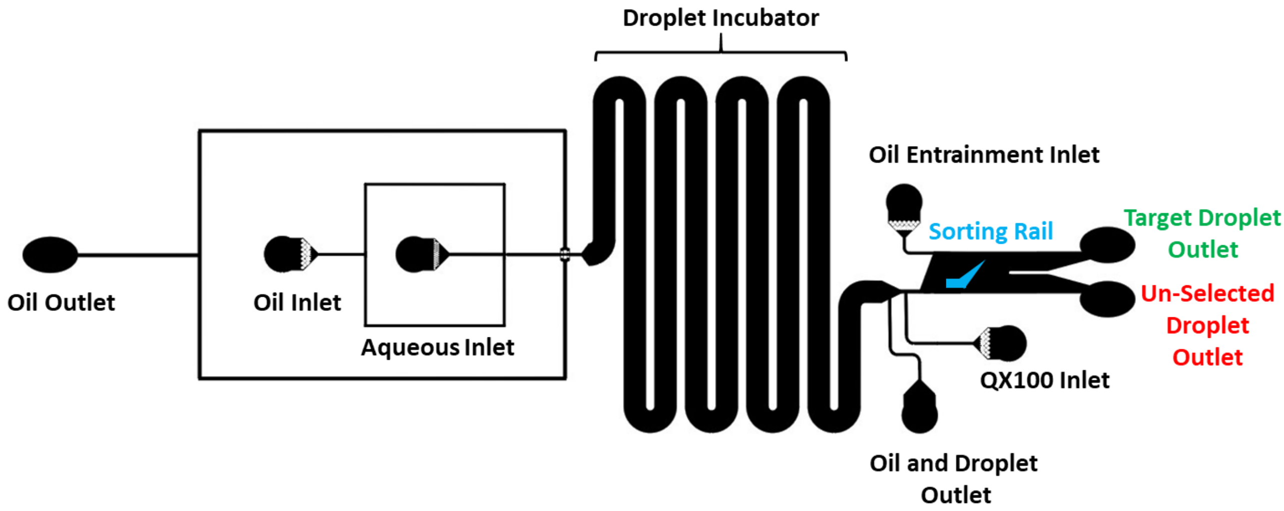

Supplement: Supplementary file 1 [file micromachines-11-00964-s001.zip › micromachines-956036/micromachines-956036-sup.pdf]
